# Vitamin B_12_ – a scoping review for Nordic Nutrition Recommendations 2023

**DOI:** 10.29219/fnr.v67.10257

**Published:** 2023-11-08

**Authors:** Anne-Lise Bjørke-Monsen, Vegard Lysne

**Affiliations:** 1 Department of Medical Biochemistry and Pharmacology, Haukeland University Hospital, Bergen, Norway;; 2 Norwegian Institute of Public Health, Oslo, Norway

**Keywords:** Vitamin B_12_, cobalamin, one-carbon metabolism, homocysteine, methyl malonic acid, nutrition recommendations

## Abstract

Vitamin B_12_ (cobalamin) is essential for normal metabolic function, and even moderate deficiency of this vitamin has negative health effects. Vitamin B_12_ is found in animal foods, and as vegetarian diets are increasingly popular in Western countries, one might expect a higher prevalence of vitamin B_12_ deficiency in the Nordic population. Setting recommendations for vitamin B_12_ intake has proven to be difficult, as uptake of vitamin B_12_ varies substantially, the clinical deficiency symptoms are often diffuse, and there is no clear agreement on the decision limits for vitamin B_12_ deficiency. Vitamin B_12_ deficiency is reported to be particularly common among pregnant women and infants, despite the fact that less than 1% of Norwegian pregnant women have a cobalamin intake below the Nordic Nutrition Recommendations 2012-recommended level of 2.0 µg/day. In addition, the assumption that breast milk contains sufficient vitamin B_12_ for optimal health and neurodevelopment during the first 6 months of life does not comply with the high prevalence of insufficient vitamin B_12_ status in this age group. Recommended intakes of vitamin B_12_ vary among age groups and must be based on markers of cobalamin status, indicating an optimal intracellular biochemical status, and not merely absence of clinical signs of vitamin B_12_ deficiency.

## Popular scientific summary

- Vitamin B_12_ (cobalamin) refers to a group of cobalt-containing compounds, corrinoids, that are biologically active in humans.
- Vitamin B_12_ is essential for normal development, metabolic and neurological function, and blood formation.
- Vitamin B_12_ only occurs in animal foods or fortified foods.
- Since biomarkers for vitamin B_12_ status have different limitations, recommendations should be based on a combination of biomarkers.
- There is no consensus regarding what levels of vitamin B_12_ biomarkers should be considered optimal.

The aim of this scoping review is to describe the totality of evidence for the role of vitamin B_12_ for health-related outcomes as a basis for setting and updating dietary reference values (DRVs) for the Nordic Nutrition Recommendations (NNR) 2023 (Box 1). Vitamin B_12_ is the common term for a group of cobalt-containing compounds (corrinoids) that are biologically active in humans. Cobalamin is used synonymously with vitamin B_12_.

Vitamin B_12_ has two metabolic functions. Methylcobalamin serves as cofactor for methionine synthase, involved in the transfer of a methyl group from folate to homocysteine, regenerating methionine. Adenosylcobalamin is a cofactor for methylmalonyl coenzyme A mutase, involved in the metabolism of odd-chain fatty acids, amino acids, and cholesterol. Insufficient activity of these enzymes, due to vitamin B_12_ deficiency, results in the accumulation of homocysteine and/or methylmalonic acid (MMA), which therefore are considered functional markers of vitamin B_12_ status (1).

Vitamin B_12_ is produced by soil bacteria and is found in animal food and soil-contaminated food (2). Vegetarian and vegan diets are increasingly popular in Western countries, particularly among young women (3). Although these diets are considered nutritionally adequate and healthy when appropriately planned, specific nutritional knowledge is needed to achieve an optimal vegetarian diet with regard to vitamin B_12_ intake and status. However, this is not always the case, even among well-educated people, and a higher prevalence of vitamin B_12_ deficiency in the Nordic population might be expected (4).

Vitamin B_12_ is essential for normal metabolic function, and even moderate deficiency of this vitamin has negative health effects, particularly in young infants, where vitamin B_12_ insufficiency is associated with impaired neurodevelopment (5–7). Vitamin B_12_ deficiency is reported to be common among Norwegian pregnant women (8) and infants (9). However, only 0.8% of Norwegian mothers in pregnancy week 18 had a vitamin B_12_ intake below the recommended intake (RI) in the NNRs 2012 of 2.0 µg/day (10), indicating that the recommendations may be too low.

Setting recommendations for vitamin B_12_ intake is difficult, and there are a number of uncertainties to consider. The absorption rate of vitamin B_12_ varies from 30 to 90%, reported estimated body stores vary from 2 to 3 mg, and daily losses range from 1.4 to 5.1 μg. The vitamin B_12_ intake needed just to compensate for daily losses is estimated to be in the range from 3.8 to 20.7 µg per day (11). In addition, there is no clear agreement on what levels of serum vitamin B_12_, plasma holotranscobalamin (holoTC), the metabolic markers plasma total homocysteine (tHcy), and serum MMA constitute an optimal vitamin B_12_ status. It is however important to remember that vitamin B_12_ is essential for normal metabolic function; as long as vitamin B_12_ is taken orally, either in food or in supplements containing a moderate vitamin B_12_ content, the body will actively regulate the uptake in ileum, and there is no evidence that intakes up to 100 µg/d from foods and supplements represent a health risk (12).

## Methods

This scoping review follows the protocol developed within the NNR2023 project (13). The sources of evidence used in the scoping review follow the eligibility criteria described previously (2).

The main literature search for this review was performed in MEDLINE on July 1st, 2022, with a following search string: (b12, vitamin[MeSH Terms] OR cobalamin[MeSH Terms]) AND review[Publication Type] AND (‘2011’[Date – Publication] : ‘3000’[Date – Publication]) AND Humans[Filter]. The number of hits was 453. Based on the title and abstract, a total of 32 articles were picked up, of which 18 were considered as relevant (11, 14–30). Three additional articles were identified through a supplementary search (31–33). We also identified relevant literature for this scoping review via ‘snowballing’/citation chasing that was relevant for the background information. No published articles were considered by the committee as a qualified systematic review (34), and a de novo NNR2023 systematic review was published in 2023 (35).

## Physiology

Vitamin B_12_ refers to a group of cobalt-containing compounds (corrinoids) that are biologically active in humans. Corrinoids also include vitamin B_12_ analogs, which are not biologically active, such as cobamides and cobinamides. The vitamin B_12_ molecule consists of a cobalt-containing corrin ring, a 5,6-dimethylbenzimidazole base, a sugar, and an aminopropanol group. A functional ligand is covalently bound to the cobalt at the upper axial position, which can either be a methyl-, adenosyl-, cyano-, or hydroxyl group.

### Absorption and intestinal transport

Dietary vitamin B_12_ is absorbed via an active receptor-mediated physiological process and via a less efficient passive diffusion pathway. In the acidic environment of the stomach, dietary protein-bound vitamin B_12_ is cleaved from the protein by gastric enzymes and bound to salivary-derived haptocorrin (HC). Stimulated by food intake, intrinsic factor (IF) is produced by the parietal cells in the stomach, and both IF and the haptocorrin-vitamin B_12_ complex are transported to the small intestine. In the less acidic environment of the upper small intestine, vitamin B12 is released from HC, and the free vitamin B_12_ binds to IF. While HC binds all corronoids, IF for all practical purposes only recognizes and binds biologically active cobalamins.

*Box 1. *The Nordic Nutrition Recommendations (NNR) 2023 project

- This paper is one of many scoping reviews commissioned as part of the Nordic Nutrition Recommendations 2023 (NNR2023) project (13).
- The papers are included in the extended NNR2023 report, but, for transparency, these scoping reviews are also published in Food & Nutrition Research.
- The scoping reviews have been peer reviewed by independent experts in the research field according to the standard procedures of the journal.
- The scoping reviews have also been subjected to public consultations (see report to be published by the NNR2023 project).
- The NNR2023 committee has served as the editorial board.
- While these papers are a main fundament, the NNR2023 committee has the sole responsibility for setting dietary reference values in the NNR2023 project.

The IF-vitamin B_12_ complex is transported to the distal ileum, where it binds to the cubam receptor and enters the enterocytes through endocytosis. The availability of cubam receptors is the rate-limiting factor for receptor-mediated absorption of cobalamin. It has been reported that the fractional absorption decreases with increasing dose (14). Bioavailability studies have demonstrated that while about 50% of a 1 µg oral dose is absorbed, only 5% of a 25 µg dose is retained. Furthermore, during the subsequent 4–6 h, active uptake is restricted due to regeneration of cubam receptors (14). In total, a maximum of ~2 μg vitamin B_12_ can be absorbed from a meal through the active route. Additionally, approximately 1.2% of vitamin B_12_ is absorbed passively (36). Normally, this accounts for a small proportion of total vitamin B_12_ absorption, but becomes relevant if vitamin B12 is consumed in high doses (>50 µg). Bioavailability of vitamin B_12_ from various foods, as assessed by whole-body retention or fecal excretion, ranges from about 20% up to 90% at single doses of 0.25 µg to 5 µg (37). It is estimated that approximately 50% of dietary vitamin B_12_ is absorbed by healthy adults with normal gastric function (38, 39).

### Transport in blood and enterohepatic circulation

Upon entering the enterocyte, IF is degraded, and free vitamin B_12_ is released into the cytosol and transported into the circulation through multidrug resistance protein 1 (MRP1). In the circulation, vitamin B_12_ is transported bound to either HC or transcobalamin (TC). About 70–90% of circulating vitamin B_12_ is bound to HC, which can be taken up by the liver through specific receptors. About 20–30% of circulating vitamin B_12_ is bound to TC (holoTC), which is responsible for delivering vitamin B_12_ to all tissues through endocytosis facilitated by the transcobalamin receptor (TCR) (14). HC-bound vitamin B_12_ has a half-life of several days compared to the half-life of about an hour for holoTC (40). HC-bound vitamin B_12_ and vitamin B_12_ analogues are excreted from the liver through bile and cleaved from HC in the intestines. While the analogues are excreted in feces, vitamin B_12_ may be reabsorbed, referred to as the enterohepatic circulation.

### Intracellular metabolism

Once bound to the TCR on the target cells membrane, the holoTC-TCR complex is taken up into the cell by endocytosis. Within the lysosome, vitamin B_12_ is released, the TC is degraded, and the TCR is recycled back to the cell surface. A series of vitamin B_12_ processing proteins facilitates the transport out of the lysosome (CblF/CblJ), removal of the upper axial ligand (CblC/CblX), partitioning to the cytosolic or mitochondrial compartment (CblD), and the formation of the two active cobalamin cofactors methylcobalamin (CblE/CblG) and adenosylcobalamin (CblA/CblB/CblH/mut) (1).

### Molecular functions

Vitamin B_12_ is a cofactor for only two enzymes in the human metabolism (2). Methylcobalamin is a cofactor for methionine synthase – the enzyme that catalyzes the conversion of homocysteine to methionine. This reaction requires the transfer of a methylgroup from 5’-methyl tetrahydrofolate. Intracellular deficiency of folate and/or vitamin B_12_ therefore results in increased plasma concentrations of tHcy. Adenosylcobalamin is a cofactor for methylmalonyl-CoA mutase in the isomerization of methylmalonyl-CoA to succinyl-CoA. Vitamin B_12_ insufficiency therefore results in increased serum MMA concentrations (2).

### Specific groups

#### Infants, children, and adolescents

The vitamin B_12_ status in the new-born is a result of maternal status during pregnancy, gestational age, and birth weight. Infants who are born premature or with a low birth weight (LBW) or with a vitamin B_12_ deficient mother are prone to develop vitamin B_12_ deficiency during the first weeks of life, something that may impair neurodevelopment (5, 9). During the first weeks of life, there is a considerable decrease in serum cobalamin, accompanied by a marked increase in plasma tHcy and MMA (41–43). The lowest vitamin B_12_ levels and the highest plasma tHcy and MMA levels in childhood are seen in infants 6 weeks to 6 months of age. In older children (>6 months), serum vitamin B_12_ increases and peaks at 3–7 years and then decreases, and median plasma tHcy remains low (<6 μmol/L) and increases from the age of 7 (43, 44).

#### Pregnancy and lactation

All markers of vitamin B_12_ status are reduced in pregnant compared to non-pregnant women (8). Serum vitamin B_12_ decreases during pregnancy. This is accompanied by a gradual increase in the metabolic markers, indicative of a functional intracellular vitamin B_12_ depletion, despite the fact that both plasma tHcy and MMA are substantially reduced compared to non-pregnant women (8).

Serum vitamin B_12_ is increased by more than 40% at 6 weeks postpartum and remains so as long as the mothers are lactating. An additional moderate increase in the metabolic markers plasmas tHcy and MMA is observed, suggestive of maternal intracellular vitamin B_12_ depletion, despite high serum concentrations (8). Vitamin B_12_ concentrations in breast milk decrease during the lactational period with the highest levels seen in the first 4 weeks (45).

### Inborn errors of metabolism

There are several inborn errors of vitamin B_12_ metabolism, which can be roughly divided into genetic mutations affecting absorption and transport, and mutations affecting intracellular vitamin B_12_ processing. The former category includes Intrinsic factor deficiency and Immerslund-Gräsbeck syndrome, which inhibits absorption either through the lack of intrinsic factor or a defect in the cobalamin receptor facilitating intestinal uptake, ultimately resulting in vitamin B12 deficiency. Haptocorrin deficiency is considered benign and results in low serum vitamin B_12_ and normal concentrations of the metabolic markers. The inborn errors affecting intracellular processing are categorized based on whether they result in accumulation of Hcy and/or MMA. CblF, CblJ, CblC, CblX, or CblD are characterized by increased levels of both tHcy and MMA, and CblE and CblG yield isolated hyperhomocysteinemia, whereas CblA and CblB yield isolated methylmalonic aciduria. Most inborn errors of vitamin B_12_ metabolism are rare, and the most common mutation is CblC, which has been reported in >500 patients (46).

## Assessment of nutrient status

Vitamin B_12_ status is judged based on the estimated dietary vitamin B_12_ intake and relevant symptoms that may suggest vitamin B_12_ deficiency or insufficiency. Biomarkers of vitamin B_12_ status include serum vitamin B_12_ and holoTC (bioavailable fraction in the circulation), while the functional biomarkers, plasma total tHcy and MMA, reflect intracellular vitamin B_12_ function. Because B_12_ is essential for folate metabolism, it is also important to consider folate status.

Many laboratories still use the 2.5 percentile, ranging from 145 to 200 pmol/L for serum vitamin B_12_, to define B_12_ deficiency. A reference interval is typically defined as the 95% interval between the lower 2.5th and the upper 97.5th percentiles derived from the distribution of results from an apparently healthy reference population (47). Reference intervals merely describe the vitamin B_12_ status in a specific population and will differ according to the diet in the tested population. One need clinical decision to define deficiency.

Clinical decision limits define a value above or below a threshold associated with a significantly higher risk of adverse clinical outcomes or diagnostic for the presence of a specific disease (47). Vitamin B_12_ deficiency associated with classic hematological and neurological manifestations is relatively uncommon, and as clinical symptoms of vitamin B_12_ deficiency generally are subtle and difficult to diagnose, a diagnostic delay of several months is common among infants, children, and adults (2, 5), something which is particularly unfortunate in young infants.

Various algorithms have been proposed in order to separate vitamin B_12_ insufficiency from deficiency (2); however, on the cellular levels, there is a physiologic transition from inadequate to clearly deficient states of vitamin B_12_. In both children and adults, the metabolic markers tHcy and MMA start to increase when serum vitamin B_12_ falls below ~500–550 pmol/L, indicating suboptimal intracellular vitamin B_12_ stores, with a steeper increase in both markers when serum vitamin B_12_ falls below 250–275 pmol/L, indicating biochemical deficiency (48, 49). Studies in humans show that biomarkers for DNA damage, such as hypomethylation, chromosome breaks, uracil incorporation, and micronucleus formation, are minimized when serum concentration of vitamin B_12_ is greater than 300 pmol/L (50), indicating that also vitamin B_12_ insufficiency is harmful and should be treated. It is obviously not good medical practice to wait until the store of an essential micronutrient is depleted.

In the following, we will comment on the individual biomarkers and the biological variations that should be taken into account when employing them. All four vitamin B_12_ biomarkers have limitations as standalone markers, and there is currently no clear agreement on the decision limits for any of these parameters concerning vitamin B_12_ deficiency or insufficiency (1, 2). Current diagnostic practice may be too complicated and focused on separating insufficiency from deficiency. As vitamin B_12_ is an essential micronutrient it is also important to focus of what is an adequate status.

### Serum or plasma cobalamin

Serum vitamin B_12_ is still the primary marker of vitamin B_12_ status among children, adults, and pregnant women (2). The assays used to analyze human samples are widely available, cheaper than all the other vitamin B_12_ markers, and specific for biologically active cobalamins. A limitation of this biomarker is that it measures the total circulating cobalamin, of which the majority (70–90%) is bound to haptocorrin, which may be affected by high estrogen levels, cancer, and genetically low haptocorrin levels. In these circumstances, serum vitamin B_12_ concentrations may not accurately reflect intracellular vitamin B_12_ status (1, 2).

The serum concentrations are not substantially affected by recent dietary intake, meaning prandial status is not a major concern when collecting samples. In the longer term (months), the concentrations increase with increasing habitual dietary intake and have been reported to plateau at vitamin B_12_ intakes of 7–10 µg (14). A meta-analysis of 37 randomized controlled trials (RCTs) and 19 observational studies aimed to quantify the dose-response relationship and reported that each doubling of vitamin B_12_ intake increased circulating concentrations by 11% (95% CI 9.4–12.5%). However, substantial heterogeneity between the studies, not explained by study design, age, or vitamin B_12_ dose, warrants caution when interpreting the reported associations (15).

Diagnostic cutoffs vary from below 148 up to 300 pmol/L depending on the outcome (2). The lower decision levels (<148 pmol/L) are generally based on the presence of clinical deficiency symptoms, while studies show that DNA damage is minimized when serum vitamin B_12_ is >300 pmol/L (50). A steep increase in both plasma tHcy and MMA occurs when serum vitamin B_12_ falls below 250–275 pmol/L, indicating biochemical insufficiency and a cellular need for vitamin B_12_.

All vitamin B_12_ markers are decreased during pregnancy, so specific decision limits must be used. A maternal vitamin B_12_ concentration >275 pmol/L (measured by immunoassay) in week 18 of pregnancy has been recommended to secure an optimal infant vitamin B_12_ status for the first 6 months of life (8, 51).

### HoloTC

Circulating holoTC represents the active fraction (10–30%) of serum vitamin B_12_, and the test has been shown to provide a somewhat better or an equal discrimination compared to serum vitamin B_12_, also depending upon the decision limits used (52). Unlike the other biomarkers, HoloTC increases in the postprandial period and is therefore a better indicator of recent intake (14). This is taken advantage of when evaluating vitamin B_12_ absorption with the CobaSorb test, measuring the holoTC response after an oral cyanocobalamin dose (53). Circulating concentrations also increases with increasing habitual vitamin B_12_ intakes and stabilizes at intakes >4 µg (14, 54).

HoloTC is reported to be affected by genetic polymorphisms (55) and renal function (56). There is a lack of consensus concerning decision limits with current suggested limits for deficiency in adults <35–40 pmol/L, based on the relation between holoTC and serum MMA concentration (14). While the other biomarkers of vitamin B_12_ status change during pregnancy, holoTC has been reported to be unaffected (14). However, plasma holoTC concentrations are reported to be reduced by 24% from preconception to pregnancy week 20, with an additional 17% reduction at labor (57). As long as holoTC decision limits for pregnant women and children are missing, serum vitamin B_12_ remains the preferred biomarker in these groups.

### Plasma MMA

Plasma MMA also accumulates with insufficient intracellular vitamin B_12_ status, due to reduced activity of methylmalonyl-CoA mutase. As this reaction is not directly influenced by other micronutrients, MMA has, for a long time, been considered the most specific and informative of the vitamin B_12_ biomarkers. However, MMA concentrations are influenced by several other factors. Less than 17% of the MMA concentrations in blood are determined by cobalamin, renal function, age, and gender, and MMA is therefore not the perfect vitamin B_12_ marker (49). Currently, higher concentrations are seen with impaired renal function and with increasing age, especially after 70 years (49). The latter is not entirely explained by age-related decline in kidney function. Furthermore, two genetic variants have been reported to explain 12% of the variance in circulating MMA (58). In a meta-analysis, a 7% decrease (95% CI 4–10%) in MMA was reported for each doubling in vitamin B_12_ intake (15).

In adults with normal kidney functions, clinical decison limits in the range >0.376 to >0.271 μmol/L have been suggested as markers for vitamin B_12_ deficiency. In order to rule out impaired renal function as the cause of elevated MMA, renal function should be evaluated (14).

MMA has been reported to increase throughout pregnancy, indicating vitamin B_12_ insufficiency (8, 59). However, compared to non-pregnant women, lower concentrations have been reported in pregnant women (8). In infants and toddlers, the MMA concentrations are higher across the entire vitamin B_12_ spectrum compared to older children and adults (43), in this age-group tHcy should be used as a vitamin B_12_ marker. In older children age-specific decision limits should be used.

### Plasma tHcy

Plasma tHcy accumulates with insufficient intracellular vitamin B_12_ status, due to reduced function of methionine synthase, referred to as hyperhomocysteinemia. Plasma tHcy concentrations are not a specific vitamin B_12_ status marker, as it also increases with insufficient intakes of folate and to a lesser degree with low intake of vitamin B6.

Plasma tHcy concentrations increase with age and decreased renal function and are influenced by sex and pregnancy, so it is necessary to use age-specific decision limits (2). The most common decision limit to define hyperhomocysteinemia is >15 µmol/L in adults.

Plasma tHcy is the preferable metabolic marker for vitamin B_12_ status in infants and toddlers, whereas in older children and adults, tHcy is primarily a folate marker, and MMA is the preferable vitamin B_12_ marker (2). In infants and toddlers up to 3 years, a plasma tHcy concentration >6.5 μmol/L indicates vitamin B_12_ deficiency (41, 44).

## Dietary intake in Nordic and Baltic countries

Vitamin B_12_ only occurs in foods of animal origin and in fortified foods. Meat, liver, eggs, dairy products, fish, and shellfish are particularly good sources and are the main vitamin B_12_ sources in the average diet.

The average vitamin B_12_ intake in the Nordic countries, according to national dietary surveys, ranged from 4.0–6.4 to 5.5–8.9 µg per day in adult women and men, respectively (60).

### Vegans/Vegetarians

Vegetarian and, especially, vegan diets tend to contain low or no amounts of vitamin B_12_, and these diets are associated with an increased risk of vitamin B_12_ deficiency unless adequate vitamin supplementation is implemented (23, 61–64). Many vegan organizations have set detailed instructions for taking a supplement, and the main principle is that the less often the vitamin B_12_ supplement is taken, the greater the dose must be; non-prescription vitamin B_12_ supplements containing up to 1,000 µg vitamin B_12_ per tablet are currently available in pharmacies.

Plant foods might contain trace amounts due to bacterial or soil contamination or as a result of fermentation. The amounts of vitamin B_12_ in seaweeds cannot be considered a reliable or relevant source of vitamin B_12_ for humans (65). Some plant-based milk substitutes are fortified with vitamin B_12_ and can be an important source of vitamin B_12_ in vegans. The nutritional quality of plant-based meat analogues on the Swedish market was assessed and published in 2022. The authors showed that vitamin B_12_ contents in fortified products differed and were lower, similar, or higher compared to meat references (66).

A recent study of vitamin B_12_ status in Norwegian vegans and vegetarians showed that average daily supplement use and older age were predictors of higher serum B_12_ concentrations. In this study, none of the participants were considered to be B_12_ depleted; however, low serum B_12_ concentration was found in 14% of the participants (67).

### Pregnant women

In pregnancy week 18, women from the Norwegian Mother, Father and Child Cohort Study (MoBa) had a median vitamin B_12_ intake of 8.5 µg/day from diet and supplements (10).

### Vitamin B_12_ in breast milk and formula and intake in infants

In breast milk from Danish mothers, most of them took daily multivitamin supplements containing 1.0–4.5 μg cobalamin, the median (25^th^ to 75^th^ percentile) vitamin B_12_ concentration at 2 weeks was 0.76 (0.21–1.88) nmol/L, at 4 months was 0.29 (0.14–0.69) nmol/L, and at 9 months was 0.44 (0.16–1.94) nmol/L (45). In breast milk from Norwegian mothers, users of supplements versus non-users, the median (25^th^ to 75^th^ percentile) vitamin B12 concentration at 6 weeks was 0.34 (0.23–0.46) versus 0.29 (0.16–0.36) nmol/L, at 4 months was 0.31 (0.21–0.40) versus 0.27 (0.18–0.48) nmol/L, and at 6 months was 0.35 (0.22–0.54) versus 0.24, (0.19–0.47) nmol/L. Mean vitamin B_12_ content in 11 different formula milk was 2.2 µg/L in a Danish study published in 2016 (68). Table 1 shows the estimated vitamin B_12_ intake in infants from breast milk from these Danish and Norwegian mothers and from formula milk. Based on these data, formula-fed infants receive from 2.1 (at 2 weeks) up to 5.2 (at 4 months) times more vitamin B_12_ per day compared to breast-fed infants.

**Table 1:** Median (IQR) vitamin B_12_ intake from breast milk versus formula milk in infants

Parameters		2 weeks	6 weeks	4 months	6 months	9 months
Mean weight, kg		3.7	4.8	6.7	7.6	8.6
Mean milk intake/day		150 ml/kg	130 ml/kg	120 ml/kg	120 ml/kg	100 ml/kg
Median infant vitamin B_12_ intake from:						
Danish mothers, +Suppl. (45)		0.57 µg/day		0.31 µg/day		0.51 µg/day
Norwegian mothers (8)	+Suppl.		0.29 µg/day	0.34 µg/day	0.43 µg/day	
	−Suppl.		0.24 µg/day	0.29 µg/day	0.30 µg/day	
Formula (68)		1.22 µg/day	1.37 µg/day	1.77 µg/day	2.00 µg/day	1.89 µg/day

Despite a higher milk intake with increasing age, daily vitamin B_12_ intake is reduced from 2 weeks to 6 months in exclusively breast-fed infants (8, 45, 69, 70).

In a Norwegian study, the estimated vitamin B_12_ intake at 12 months was median 1.7 (IQR 1.1–2.2) μg per day from solid food and breastmilk versus 2.3 (1.8–3.0) μg per day from solid food and formula (70).

### Infants born premature or with LBW

Infants born premature or with a low birthweight have smaller vitamin B_12_ stores and an increased risk of deficiency during the first year of life (71). However, as many premature and LBW infants have problems breastfeeding, this group is often mainly formula fed, something which will increase their vitamin B_12_ intake and improve their status (7). In exclusively breast-fed premature or low birthweight infants, one must be aware of development of vitamin deficiency.

### Elderly

Dietary surveys in the Nordic countries suggest that vitamin B_12_ intake in the elderly is slightly higher compared to younger adults, with estimated intakes ranging from 5.2–6.6 to 5.2–10.8 μg/day in women and men, respectively. However, with increasing age, the production of both hydrochloric acid and intrinsic factor declines, putting elderly at increased risk of developing vitamin B_12_ deficiency or insufficiency (2). It has been estimated that 10–30% of elderly have some form of vitamin B_12_ malabsorption due to atrophic gastritis, which is often undiagnosed.

## Health outcomes relevant for Nordic and Baltic countries

### Deficiency

There is currently no clear agreement on what symptoms constitute clinical vitamin B_12_ deficiency and what decision limits for vitamin B_12_ status one should use (2), but one guiding principle is that if the symptoms are alleviated by treatment with cobalamin, then it is likely that there was a degree of vitamin B_12_ insufficiency. An adequate supply of vitamin B_12_ is essential for normal development, neurological function, and blood formation (2). Vitamin B_12_ deficiency in adults may result in neurological and psychiatric symptoms and/or macrocytic and megaloblastic anemia (2, 72, 91). Lindenbaum et al. reported vitamin B_12_ deficient patients with psychiatric symptoms, but without anemia or macrocytosis (72). The reason why some patients mainly present with megaloblastic anemia and others with neurologic symptoms remains unknown. Neurological manifestations, such as numbness and paraesthesia in the extremities; loss of position and vibratory sensation; difficulty walking; depression and irritability; diminished cognitive function; and psychosis may be observed in the absence of hematologic disease, (72, 73). Subclinical deficiency detected by metabolic markers i.e. increased tHcy and MMA homocysteine and MMA, which are predictors of developmental and degenerative diseases, is much more common in the general population (50). The observed biochemical insufficiency at B_12_ levels below ~500–550 pmol/L raises the question whether such vitamin B_12_ insufficiency has clinical consequences (48). A review has shown a high prevalence of vitamin B_12_ insufficiency in the world and has identified several examples of adverse clinical outcomes, mainly in relation to functions of the nervous system (74). Others have suggested that vitamin B_12_ deficiency/insufficiency is related to increased prooxidant and reduced antioxidant status (32).

#### Vitamin B_12_ deficiency during pregnancy and infancy

Vitamin B_12_ deficiency in women of fertile age is associated with infertility, early pregnancy loss (75), increased prevalence of preeclampsia, preterm birth (76) possibly LBW (27, 29), and a moderate risk of neural tube defects (77). A review published including 23 studies from 1961 to 2017 reported a positive effect of vitamin B_12_ on semen quality, including increasing sperm count, enhanced sperm motility, and reduced sperm DNA damage (78).

Maternal vitamin B_12_ deficiency during pregnancy is associated with vitamin B_12_ deficiency in the infant (8). Symptoms of vitamin B_12_ deficiency in children differ with age, presenting a continuum from subtle developmental delay to life-threatening clinical conditions. The first months of life is the most rapid period of brain development, with a brain growth ranging per day from 1% per day and gradually decreasing to 0.4% at 3 months (79). An optimal micronutrient status is particularly important during this period. In infants, symptoms of vitamin B_12_ deficiency include irritability, failure to thrive, apathy, anorexia, gastrointestinal reflux, constipation, and developmental delay and regression (5, 6), all of which respond remarkably and rapidly to supplementation (6). Symptoms of vitamin B_12_ deficiency are difficult to detect in all age groups, but particularly in infants, and there tends to be a diagnostic delay up to 4 months in this age group (5, 9). Several reports show that even moderate deficiency in children may be harmful, and long-term consequences of neurological deterioration may persist after vitamin B_12_ deficiency has been treated (9). Even in well-nourished children, a low vitamin B_12_ concentration has been associated with a lower score for psychomotor development. A randomized controlled vitamin B_12_ intervention study in infants with moderate B_12_ demonstrated significantly improved mootor development in only 3 weeks (6). In Danish children aged 36 months with a mean (SD) serum vitamin B_12_ concentration of 653 (240) pmol/L, an increase of 100 pmol/l in serum vitamin B_12_ corresponded with a 1.5 increase in total Ages and Stages Questionnaire (ASQ) score, a measure of psychomotor development, raising a question about what is the optimal serum vitamin B_12_ concentration in young children. Children in the lowest vitamin B12 quartile had about 10 ASQ scores lower compared to the other quartiles (80).

### Toxicity

As long as vitamin B_12_ is taken orally, either in food or in supplements containing a moderate vitamin B_12_ content (<50 µg), the body will actively regulate the uptake in ileum, and excess vitamin B_12_ will be excreted in urine. To patients with confirmed vitamin B_12_ deficiency due to absorption problems, a peroral dose of 4,000 cyanocobalamin µg daily for 1 month, followed by 1–2,000 µg per day, is now recommended as an alternative to vitamin B_12_ injection therapy. There are intake known severe adverse effects of excess vitamin B_12_ (12). Potential negative effects of megadoses, i.e. on the microbiome, are largely unknown, but one study reported that vitamin B_12_ supplementation may give acne development due to changes in the skin microbiome (81).

### Obesity

The association between circulating vitamin B_12_ concentrations and the body mass index (BMI) was investigated in a systematic review of 19 observational studies including 7,055 participants (33). Overall, it was reported that those with BMI categorized as overweight or obese had mean 21–56 pmol/L lower serum vitamin B_12_ concentrations compared to those with normal weight. In meta-regression analyses, no inverse or J-shaped association was established, but the authors concluded that the results from the direct pairwise comparisons supported further investigation on the association between vitamin B_12_ status and overweight/obesity. Lower vitamin B_12_ levels with higher BMI are also reported in pregnant women (82).

### Cardiovascular disease (CVD) and diabetes type 2

In a Cochrane review of 15 RCTs, Marti-Carvajal et al. reported on the effect of homocysteine-lowering intervention with B-vitamin supplements (vitamin B_6_, folate, and vitamin B_12_ alone or in combination) on cardiovascular disease outcomes and mortality (20). Compared to placebo, they reported reduced risk of stroke (RR [95% CI] 0.90 [0.82–0.99]), but no effect on myocardial infarction risk (1.02 [0.95–1.10]) or all-cause mortality (1.01 [0.96–1.06]). Rafnsson et al. performed a systematic review of 7 cohort studies found only very limited evidence for the associations between low serum vitamin B_12_ and increased risk of mortality and morbidity from either cardiovascular diseases or type 2 diabetes in adults (26).

### Cancer

Observational studies have found an association between higher circulating vitamin B_12_ concentrations and increased risk of prostate (83) and total cancers (84), and a high serum vitamin B_12_ has been proposed as a cancer biomarker (85). The elevated vitamin B_12_ levels appear to be due to increased haptocorrin concentrations and not to raised holoTC (83, 86). Furthermore, clinical trials do not support an association between higher vitamin B_12_ intakes and cancer risk (87–89). A meta-analysis of 18 RCTs including approximately 75,000 individuals found that supplements containing B vitamins, including 20 to 2,000 µg/day vitamin B_12_, had little or no effect on cancer incidence, cancer deaths, or all-cause mortality (88). A dose-response meta-analysis including data from 10,600 patients suggested an inverse association between both total and dietary vitamin B_12_ intakes and colorectal cancer risk (90). This was supported in a meta-analysis of 13 case-control studies, reporting an inverse association between circulating vitamin B_12_ and colorectal cancer risk (not statistically significant), and a positive association with circulating tHcy (30). However, a meta-analysis of 10 observational studies with 3,164 cases observed a higher risk of esophageal cancer when comparing the highest vs lowest intake (OR [95% CI] 1.30 [1.05–1.62]), and a linear dose-response per 1 µg/d increment (1.02 [1.00–1.03]) (25).

### Osteoporosis and bone health

The association of vitamin B_12_ status with bone mineral density and osteoporotic fractures was investigated in a systematic review by Macedo et al., including 6 longitudinal studies, 9 cross-sectional studies, and two RCTs (19). The authors concluded that the potential effect of vitamin B_12_ deficiency on bone health and the underlying mechanisms warrant further research, especially in vulnerable groups such as postmenopausal and elderly women.

### Mental health

Whereas most studies question the effect of vitamin B_12_ on mental helth in the elderly, positive associations are reported in children (80). Data on vitamin B_12_ status and associated cognitive function and depression in elderly people are conflicting. A Cochrane review from 2003 found that the evidence for any effect of vitamin B_12_ on improving the cognitive function of people with dementia and low serum vitamin B_12_ levels was insufficient (92). The same conclusion was reached in a systematic review and meta-analysis of 19 RCTs (16), concluding that supplementation of cobalamin, vitamin B_6_, or folic acid alone, or in combination, did not appear to improve cognitive functions in individuals with or without cognitive impairment at baseline. The same authors performed an updated systematic review of 31 RCTs, and in a meta-analysis of 12 RCTs using the Mini Mental State Examination, they observed no meaningful effect in individuals with, and no effect in individuals without, cognitive impairment at baseline (31). Accordingly, there is limited evidence from observational studies to suggest an association of vitamin B_12_ status with cognitive decline or dementia in elderly people (21, 93). In a systematic review of observational studies, the authors concluded that there was insufficient evidence to establish an overall association, but it was highlighted that all four studies that used more specific biomarkers of vitamin B_12_ status (MMA and holoTC) showed consistent associations between poor vitamin B_12_ status and increased risk of cognitive decline or dementia diagnosis (22). Another systematic review identified poor vitamin B_12_ status as being associated with increased risk of vascular dementia, primarily based on cross-sectional studies (24). Treatment with a combination of folic acid and vitamin B_12_ has also shown cognitive benefits in people with mild cognitive impairment (94, 95).

Vitamin B_12_ insufficiency has been found to increase the risk of depression in the elderly (96), and a recent review concluded that early vitamin B_12_ supplementation can delay the onset of depression and improve the effect of anti-depressants (97). On the other hand, a systematic review of 11 RCTs concluded that short-term treatment with folic acid and/or vitamin B_12_ did not improve depressive symptoms. However, the results suggested that longer-term treatment may decrease the risk of relapse or onset of symptoms in those at increased risk (98). A systematic review pooling data from 10 observational studies reported consistently lower circulating vitamin B_12_ concentrations in patients with Parkinson’s disease (overall standardized mean difference [95% CI] -0.38 [-0.51, -0.25]) (28). However, no association was observed for dietary vitamin B_12_ intake and Parkinson’s disease risk.

### Eye disease

In a systematic review and meta-analysis of 11 case-control studies, including 1,072 cases, Huang et al. reported that tHcy was higher (mean difference [95% CI] 2.67 [1.6–3.74] µmol/L), and serum vitamin B_12_ was lower (64.2 [19.3–109] pmol/L) in age-related macular degeneration (AMD) cases compared to controls (17). Another systematic review and meta-analysis by Li et al. included 9 studies reporting on the serum vitamin B_12_ levels and different types of glaucoma (18). In random-effect meta-analyses, they reported no statistically significant differences in serum vitamin B_12_ between cases and controls.

## Requirement and RIs

The RI of vitamin B_12_ published in NNR 2004 and 2012 was largely based on a study published in 1958, where 20 patients with pernicious anemia were treated with an intramuscular dose of 0.5–2.0 µg vitamin B_12_ per day, and the outcome parameter was normalizing of hematological status (99). An average physiological requirement of vitamin B_12_ was set at 0.7 μg/day, based on this and other studies (38, 100). With correction for absorption efficiency (50%), the average requirement (AR) was set at 1.4 μg/day for adults. By assuming a CV of 15% and adding two SD to allow for individual variation, the RI for adults was set at 2 μg/day. The same recommendations applied to elderly and pregnant women, while an additional 0.6 µg/day was recommended to lactating women. The recommendations for children were based on an assumed requirement of 0.05 µg/kg body weight/day, and the RIs were set at 0.8 (2–5y), 1.3 (6–9y), and 2.0 (10–13y) µg/day. The NNR2012 RI was unchanged from the recommendations in NNR2004.

The NNR2012 DRVs were comparable to most other vitamin B_12_ intake recommendations by authoritative scientific bodies worldwide (14), with one notable exception. In 2015, the EFSA Panel concluded that it is impossible to determine an AR and RI and, therefore, set an adequate intake (AI) for vitamin B_12_ at 4 μg/day for men and women >14y, based on available data on different vitamin B_12_ biomarkers (101). An additional 0.5 µg/d was added for pregnancy (AI 4.5 µg/day), and 1 µg/day was added during lactation (AI 5.0 µg/day) based on fetal accumulation of vitamin B_12_ and excretion through breast milk. For children, the AIs were extrapolated from adult values and were set at 1.5 µg/day from 7 months of age.

The *de novo* NNR2023 systematic review concluded that for all included populations (pregnancy, lactation, infants, elderly, and vegetarians and vegans), there was insufficient evidence to assess whether habitual intakes in line with the previously RIs (NNR2012) were sufficient to maintain adequate status (35). No eligible studies were identified for young adults or children other than breast-fed infants.

### Adults

Daily vitamin B_12_ losses in apparently healthy adults and elderly probably range from 1.4 to 5.1 μg. A systematic review estimated that the vitamin B_12_ intakes needed to compensate for these losses ranged from 3.8 to 20.7 μg (11). However, these estimates should be interpreted with caution, as the rate-limited absorption from a single dose was not considered. To better estimate the dose needed, both the bioavailability and the division of the dose throughout the day must be considered. Based on biomarker concentrations, it has been reported that in healthy young adults and postmenopausal women, circulating concentrations of cobalamin, homocysteine, and MMA appears to plateau at intakes of 4–10 µg/day (54, 102, 103). This suggests that previous RIs (NNR2012) may be insufficient to maintain optimal biomarker status. Based on available data on different biomarkers of vitamin B_12_ status, a vitamin B_12_ intake in the range of 5–7 μg/day seems to be adequate for adults; however, no consensus exists regarding what should be considered optimal in terms of metabolic profile.

### Elderly

In NNR2012, the RI for elderly was the same as for younger adults. This is in line with the Institute of Medicine (IOM), who emphasize that because dietary vitamin B_12_ can be assumed to be poorly absorbed due to atrophic gastritis, increasing the recommendations was unlikely to be sufficient.

### Vegetarian and vegan diets

As vitamin B_12_ only occurs in foods of animal origin, vegan diets will eventually result in vitamin B_12_ deficiency unless supplemented. However, lower intake and status are also observed with less restrictive diets limiting animal food (104). It should be emphasized that symptoms of deficiency may not occur until years after adopting such diets. As repleting the body stores through supplements would take a long time, initial treatment with injections is warranted once symptoms of deficiency are present.

### Pregnancy and lactation

A maternal vitamin B_12_ concentration >275 pmol/L in week 18 of pregnancy has been recommended to ensure an adequate infant vitamin B_12_ status for the first 6 months of life (8, 49). Additionally, serum vitamin B_12_ concentration above 300 pmol/L in early pregnancy has been associated with lower incidence of neural tube defects (105).

In pregnancy week 18, women from the Norwegian MoBa study reported a median vitamin B_12_ intake of 8.5 µg/day from diet and supplements (10). Median (IQR) serum vitamin B_12_ concentration in 2,911 women from the MoBa study was 309 (249–378) pmol/L (106). This study may suggest that a vitamin B_12_ intake of 8.5 µg/d can be expected to give more than 50% of the women a sufficient vitamin B_12_ status in pregnancy week 18.

### Infants

#### Infants born at term with an appropriate weight for gestational age

The calculated AI for infants aged 0–6 months ranges from 0.3 to 0.5 μg/day and is based on the assumption that breast milk contains sufficient vitamin B_12_ for optimal health during this period of life. The data for estimated AI include an average intake of breast milk (~800 ml/d) and an average vitamin B_12_ concentration in breast milk (~0.45 μg/L).

Only 28% of infants at 6 weeks and 34% at 4 months achieve the RI of 0.4 µg per day from breast milk (8). Additionally, whether this amount of vitamin B_12_ is enough will also depend on the infant vitamin B_12_ stores, which will be low in infants born to women with vitamin B_12_ deficiency due to gastrointestinal disease or a diet with a low content of animal food. Formula-fed infants receive from 1.2 to 2 µg vitamin B_12_ per day from 2 weeks to 6 months (Table 1) and are reported to have lower plasma tHcy concentrations, indicating a better vitamin B_12_ status compared to exclusively breast-fed infants (9).

For infants aged 7–12 months, the recommended AI ranges from 0.7 to 1.5 μg/day. EFSA has the highest AI (1.5 μg/day), based on an extrapolation from the AI for adults using allometric scaling and applying a growth factor (93). At 12 months, infants with median vitamin B_12_ intake of 2.3 (25^th^–75^th^ percentile 1.8–3.0) μg per day had a lower geometric mean plasma tHcy: 4.5 (95%CI 4.1–4.9) µmol/L, compared to infants with a lower median vitamin B_12_ intake (1.7 (1.1–2.2) μg per day) plasma tHcy 5.5 (5.1–5.8) µmol/L. This study suggests that a vitamin B_12_ intake of approximately 2.3 μg per day should be sufficient for most infants aged 12 months.

#### Infants born premature or with LBW (<2,500 g)

Infants born premature or with a LBW have an increased risk of vitamin B_12_ deficiency during the first year of life (71). One study showed that breast-fed and formula-fed LBW infants had similar plasma tHcy until 20 postnatal days; after this, plasma tHcy subsequently increased in breast-fed infants, but not in formula-fed infants (107). The vitamin B_12_ intake would be 0.17 to 0.23 µg vitamin B_12_ per day (based on a vitamin B_12_ concentration in breast milk of ~0.45 µg/L and 0.4–0.5 L milk intake/day) in the breast-fed infant. A formula-fed infant would get 0.8 to 1 µg vitamin B_12_ per day (based on 2.2 µg/L vitamin B12 and 0.4–0.5 L milk intake/day) during the period from 3 to 6 weeks. As plasma tHcy did not increase in the formula-fed infants, 0.8 to 1.0 µg vitamin B_12_ per day seems to be the necessary amount of vitamin B12 intake for LBW infants aged 3 to 6 weeks.

Among Norwegian infants with a birth weight between 2 and 3 kg, those who were exclusively breast-fed for >1 month had lower vitamin B_12_ and higher tHcy levels at 4 and 6 months compared to infants who were fed formula. Additionally, the breast-fed infants had lower gross motor scores at 6 months compared to the formula-fed infants (7), indicating that even moderate vitamin B_12_ insufficiency may impair neurodevelopment. Given a vitamin B_12_ concentration in formula of 2.2 µg/L and a milk intake of 0.120 L/kg/day, the estimated vitamin B_12_ intake associated with a better metabolic status and neurodevelopment would range from 1.1 µg/day at 4 months to 1.5 µg/day at 6 months.

### Children

In healthy, non-breast-fed Norwegian toddlers at age 24 months, a plateau in serum vitamin B_12_ and holoTC was reached at a vitamin B_12_ intake of ~3 μg/day (108). Neither MMA nor tHcy concentrations decreased with increasing vitamin B_12_ intakes, which may indicate that a vitamin B12 intake of ~3 μg/day at this age secures an adequate vitamin B_12_ status.

In a survey published in 2015, Ungkost 3 (109), vitamin B_12_ intake in Norwegian children aged 9 years was mean 4.9 (SD 2.2) μg/day, and that of aged 13 years was mean 5.3 (SD 3.7) μg/day (Ungkost 3, 2015). In a study published in 2003 (110), Norwegian children aged 1–10 years had a median serum vitamin B_12_ of 551 (25th–75th percentile 456, 683) pmol/L, for the age group 10.5–15 years, it was 436 (295, 529) pmol/L, and for the age group 15.5–19 years, it was 369 (294, 452) pmol/L. As a serum vitamin B_12_ >300 pmol/L indicates an adequate vitamin B_12_ status, approximately 25% of the older children (>10.5 years) had a risk of vitamin B_12_ deficiency, whereas this was not an issue for the younger children. In Canadian children aged 6 years and adolescents, a daily consumption of vitamin B_12_ supplements was associated with higher serum vitamin B_12_ concentrations, with no additional increase in serum vitamin B_12_ at doses above 10 μg/day (111).

Based on these studies, a vitamin B_12_ intake from 3 to 4.9 μg/day may be adequate for younger children, whereas children >10 years may need a vitamin B_12_ intake in the range of 5.3–10 μg/day.
